# Global and local components of output gaps

**DOI:** 10.1007/s00181-023-02419-5

**Published:** 2023-04-13

**Authors:** Florian Eckert, Nina Mühlebach

**Affiliations:** KOF Swiss Economic Institute, ETH Zurich, Leonhardstrasse 21, 8092 Zurich, Switzerland

**Keywords:** Multi-level DFM, Bayesian state space model, Output gap decomposition, Model combination, Business cycles, Variable selection, Spatial Prior, C11, C32, C52, F44, R11

## Abstract

This paper proposes a multi-level dynamic factor model to identify common components in output gap estimates. We pool multiple estimates for 157 countries and decompose them into one global, eight regional, and 157 country-specific cycles. Our approach easily deals with mixed frequencies, ragged edges, and discontinuities in the underlying output gap estimates. To restrict the parameter space in the Bayesian state space model, we apply a stochastic search variable selection approach and base the prior inclusion probabilities on spatial information. Our results suggest that the global and the regional cycles explain a substantial proportion of the output gaps. On average, 18% of a country’s output gap is attributable to the global cycle, 24% to the regional cycle, and 58% to the local cycle.

## Introduction

Fiscal and monetary policy authorities use the output gap to determine the cyclical position of the economy, detect structural imbalances, and predict inflationary pressure (see, for instance, Gerlach and Smets 1999; Sturm and de Haan 2011; Coibion and Gorodnichenko 2015). As a result, output gaps are usually estimated at a level which corresponds to the economic aggregate that is affected by policy decisions. Typically, this corresponds to the national level. However, it is increasingly common that countercyclical policies are implemented at transnational levels. Countries might coordinate policy actions during crisis periods, or delegate them to a centralized institution if they are members of a monetary or customs union. In these instances, it is important whether cyclical imbalances are unique to one country, shared with nearby countries, or even common to all countries. This information allows policy actions to be taken at the appropriate level, thereby avoiding the issue of pro-cyclical outcomes of policy measures. For instance, in the case of a negative country-specific shock that leads to a deflationary output gap in only one particular country, it would not be efficient for a cross-country institution (for example, the central bank in a currency area) to act. By doing so, it would only contribute to imbalances in countries that are not affected by this shock. Instead, only the government of the affected country should intervene with countercyclical policies. If, on the other hand, a shock affects all countries in a region, cross-country measures taken by a centralized institution as well as coordinated interventions by several countries are probably more efficient in limiting the economic impact. Standard methods for estimating the output gap do not allow to directly identify a possible cross-country overlap of business cycles and, therefore, do not provide guidance on the level of government at which policymakers should intervene with measures to stabilize the cycle.

Having thus motivated the paper, we suggest how to deal with the problem of allocating policy action across hierarchical structures. We propose a multi-level dynamic factor model (DFM) that extracts global, regional, and local components from a large collection of output gap estimates at the country level. We build upon the multi-level factor model proposed by Kose et al. (2003). They extract common factors from macroeconomic data at different hierarchical levels. More specifically, they identify global, regional, and country-specific factors in output, consumption, and investment. Similar decomposition exercises and methodological refinements can be found in Del Negro and Otrok (2008) for GDP growth rates, Moench et al. (2013) and Kose et al. (2012) for a selection of macroeconomic variables, Bai and Wang (2015) for international bond yields, and Mumtaz and Surico (2012) for inflation rates. In a recent contribution, Berger et al. (2021) use stochastic factor selection in a multi-level DFM to test for the presence of global as well as regional cycles. We extend the literature on multi-level dynamic factor models by focusing on output gaps, thereby providing several contributions to the literature on international business cycles, output gap estimation, and the use of spatial information in model selection.

First, we extend the literature on the estimation of multi-level factor models to identify global business cycles. In contrast to the aforementioned literature, we use several output gap estimates as ‘data’ and identify the factors using linear constraints on the factor loadings. Such binary coefficient matrices are commonly used in the literature on forecast reconciliation to ensure additive coherency across hierarchical time series (see, for instance, Athanasopoulos et al. 2017; Hyndman et al. 2016; Eckert et al. 2021). Since the underlying time series share the same unit, which is the deviation of GDP from potential output in percent, all cyclical components have a clear interpretation as the additive contribution of a hierarchical level to a country’s output gap in percentage points. Furthermore, our model allows for mixed frequencies, ragged edges, and missing observations in the data. This flexibility allows us to include a large set of countries in our analysis, addressing the issue of data availability in developing countries which was highlighted, for instance, by Kose et al. (2020). The model can easily be adapted to assess whether any country aggregate shares a common cycle by changing the hierarchical structure. Aggregates are defined ex ante and can be based on geographic characteristics, currency unions, trade agreements, languages, or income levels.

Second, we provide an efficient Bayesian sampling algorithm that introduces sparsity into the large-dimensional vector autoregressive (VAR) coefficient matrices by means of stochastic search variable selection (SSVS). Since VARs involve a large number of coefficients, it is necessary to use shrinkage or selection algorithms to reduce the dimensionality of the model. For cross-sectional spatial data, LeSage and Krivelyova (1999) and LeSage and Cashell (2015) propose to shrink coefficients by incorporating spatial information in the prior specification. Their priors are based on the belief that neighboring spatial entities exert a stronger influence than non-adjacent entities. We extend their approach in several ways. Most importantly, we employ stochastic search variable selection to determine which coefficients are likely to be different from zero and should therefore be included in the regression. This approach is far more parsimonious as it only requires prior inclusion probabilities instead of normal priors. The use of prior inclusion probabilities also allows us to include spatial information in a generic way that requires little tuning. Furthermore, we propose a finer measurement for geographic proximity, namely, a continuous setup based on distances between countries instead of a binary setup based on contiguities.

Third, we use significance tests to identify meaningful business cycle fluctuations at global, regional, and local levels. Kose et al. (2020) highlight the issue that an identification of ‘global recessions’ is complicated by the fact that the commonly used definition for recessions, namely a contraction in output for at least two consecutive quarters, cannot be applied to the global level because a contraction in total global output is extremely rare. In order to address this issue, we use statistical tests to determine whether global and regional output gap components deviate significantly from zero. A comprehensive empirical study aims to quantify global, regional, and national recessions. Our data includes five different output gap estimation methods in yearly and quarterly frequency for 157 countries grouped into eight regions and covering the years 1990 to 2020. Our results suggest a strong global cycle because a substantial part of the output gap is shared with all countries. In addition, most regions exhibit a strong common business cycle. The strongest common movement exhibit the countries in North America, Europe, and East and Southeast Asia. In contrast, the regions Sub-Saharan Africa and North Africa and West Asia display almost no significant common fluctuations. On average, 18% of a country’s output gap is attributable to the global cycle, 24% to the regional cycle, and 58% to the local cycle. Furthermore, our model provides output gap estimates that are reconciled across multiple output gap estimation methods, frequencies, and countries. The reconciliation across countries is based on the assumption of co-moving output gaps across all countries. The model gives a structure in the cross-country output gaps such that the estimations borrow strength from the estimated output gaps of other countries.

The remainder of the paper is structured as follows. Section 2 presents the multi-level dynamic factor model and discusses the assumptions necessary to identify the parameters. It shows the use of stochastic search variable selection in estimating the VAR coefficients and highlights the benefits of spatial information to determine prior inclusion probabilities. Section 3 presents the data used in the empirical application and discusses the decomposition of output gaps into global, regional, and local cycles. Section 4 concludes.

## A large multi-level dynamic factor model

### Measurement

We estimate a multi-level dynamic factor model to extract common components from output gaps for various countries. It is assumed that the output gap estimates, which are measured at the local level, are noisy signals of some unobserved global, regional, and local cyclical activity. Hence, each output gap estimate is driven by a global factor that is common to all countries, a regional factor that is common only to countries in a specific region, and a country-specific factor. Since we attempt to measure the output gap using several ‘base models,’ an additional error term captures the country- and model-specific error. The choice to have a set of independently estimated output gaps is motivated by the fact that empirical researchers are often tasked with interpreting a collection of output gap estimates. A model pool is very common in output gap estimation since some estimation techniques might be more useful in certain situations and less in others. Having a set of estimates provides valuable information and redundancies.^1^

In the absence of intertemporal constraints due to mixed frequencies and ragged edges, the ‘basic’ measurement equation is given by

1 $$\begin{aligned} {{\textbf {y}}}_{t}&= \varvec{\Lambda }{{\textbf {f}}}_t + {{\textbf {e}}}_t, \qquad {{\textbf {e}}}_t \sim {\mathcal {N}}\left( {{\textbf {0}}}, \varvec{\Sigma }\right) , \end{aligned}$$

where ${{\textbf {y}}}_t$ is an *n*-dimensional vector containing output gap estimates that are subject to measurement errors. The factor loadings matrix $\varvec{\Lambda }$ is of order $n\times q$ and encodes the linear hierarchical constraints. The global, regional, and local factors are stacked into a *q*-dimensional vector ${{\textbf {f}}}_t$ that adheres to the linear constraints implied by the loadings matrix. The errors are normally distributed with mean zero and diagonal covariance matrix $\varvec{\Sigma }$. Assuming that the underlying base models aim to estimate the same unobserved output gap, the measurement errors ${{\textbf {e}}}_t$ capture model-specific estimation errors as well measurement errors in the data that enter these base models. While it is difficult to disentangle these sources of measurement errors, one might argue that it is typically the same or similar data that enters output gap estimation models. Hence, the often large dispersion in estimates is largely caused by different modeling methods, techniques, and assumptions.^2^ The assumption of uncorrelated errors is based on the fact that common economic shocks at the local level are likely to be captured by a common factor at a higher level, due to the hierarchical structure of the factors.

Such a multi-level factor model was proposed by Kose et al. (2003). In contrast to their approach, we restrict the loadings matrix $\varvec{\Lambda }$ to be binary. Therefore, the factors contribute either fully to a particular output gap estimate or not at all. From a Bayesian perspective, this specification can be interpreted as assigning zero prior probability to outcomes that do not adhere to the linear constraints. While binary factor loadings may seem restrictive compared to previous multi-level DFMs, this model choice has several advantages. First, the binary restrictions on the loadings $\varvec{\Lambda }$ identify the unobserved factors ${{\textbf {f}}}_t$ as additive contributions to a country’s output gap, measured in percentage points. This yields a straightforward interpretation of the factors as output gap components, which is unprecedented in the literature on global and local cycles. Furthermore, it allows us to perform statistical tests on the dynamic factors to determine whether the contributions of hierarchical levels to local business cycle fluctuations are significantly different from zero.

Second, the global factor contributes to the output gap of all countries. Because each output gap estimate is the sum of global, regional, and local components, we can be certain that fluctuations in the global component occur in all underlying output gap estimates. If the global factor instead only reflects the business cycle of a few countries, the question would arise whether the factor can then still be considered ‘global.’ Similarly, each regional factor contributes to the output gap of all countries within a region equally. The factor loadings serve a similar function as the summation matrix in the literature on hierarchical forecast reconciliation (see, for instance, Hyndman et al. 2011, 2016). They ensure coherency and additivity across multiple hierarchical levels.

Third, the binary restrictions resolve the scale and sign indeterminacy inherent in most factor models. If both factors ${{\textbf {f}}}_t$ and loadings $\varvec{\Lambda }$ are unobserved, infinite possibilities exist to express the same data. This observational equivalence is typically resolved using restrictions on the factor loadings (Stock and Watson 2016). Bai and Wang (2015) show that $q^2$ restrictions are necessary, typically zeros and ones in the factor loadings. Therefore, the binary restrictions proposed in this paper clearly over-identify the model, but they uniquely and coherently identify a meaningful set of underlying factors.

As a basic example, we take a collection of four countries A, B, C, and D. The countries A and B form one region, C and D form a second region. Assuming that the output gaps for each country are estimated using two methods, the variables involved in Eq. (1) are given byThe output gap for country *A*, estimated with method 1, is then given by $y_{A1,t} = f_{ABCD,t} + f_{AB,t} + f_{A,t} + e_{A1,t}$. The output gap for country *B*, estimated with method 2, is then given by $y_{B2,t} = f_{ABCD,t} + f_{AB,t} + f_{B,t} + e_{B2,t}$. Both output gap estimates contain the same global and regional components, but different local components and error terms.

The hierarchical affiliation of each country to global and local cycles is straightforward. Regional cycles, however, can be a matter of discretion and their boundaries should be determined carefully. As Mumtaz et al. (2011) and Berger and Wortmann (2020) point out, the global and group-specific factors may deviate substantially depending on the hierarchical groups chosen. In our case, the inclusion of regional aggregates serves two main purposes. First, they eliminate or reduce cross-correlation in the measurement errors by capturing comovement in the data. Second, they allow for the examination of common cyclical components in known aggregates, such as regions, currency unions, or customs unions. It is, therefore, important to determine the boundaries of the sub-aggregates such that they include homogeneous countries according to economic, political, or geographic criteria. In line with Berger et al. (2021), one might incorporate additional regional factors that are not functions of geographic proximity. In the literature on forecast reconciliation, such multi-dimensional hierarchical structures are referred to as grouped hierarchies (Wickramasuriya et al. 2019; Eckert et al. 2021). Additional regional aggregates have been suggested, for instance, by Francis et al. (2017), who endogenously determine the grouping of the countries. Their results suggest that countries with similar legal institutions and linguistic diversity share a stronger common cycle than physically close countries. Berger et al. (2021) use a stochastic search factor selection procedure and find the level of development to be a relevant factor, confirming the findings in Kose et al. (2012). However, since we test for the existence of common regional cycles, it is necessary to determine the regional aggregates exogenously and ex ante. In this study, we focus only on the spatial and regional interdependencies to examine the existence of regional business cycles.

In order to deal with certain data issues that commonly occur in empirical applications, we may wish to impose temporal linear constraints on the state equation. Output gap estimates, for instance, are usually sampled at quarterly or annual frequency. Mixed frequencies in the data can be implemented easily by specifying the factors at the highest frequency and letting low-frequency variables also load on lagged factors. In a first step, the mixed-frequency data is coerced to a matrix. All time series are converted to the highest frequency and extended to match the time of the first and last observation across the entire collection. A time series is assigned a value of zero in a specific period if there are missing observations due to publication delays, a gap in the data, or a limited history. Time series with lower frequencies are converted to the highest frequency by registering each observation in the last high-frequency entry of the corresponding low-frequency period. All other entries are filled with zeros. When mixing annual and quarterly data, for instance, the annual observation is registered in the last quarter of the corresponding year and the entries of the first three quarters are set to zero. This results in a matrix of order $T \times n$ where $t = 1, \ldots , T$ is the high-frequency time index, and *n* is the number of variables. To impose temporal linear constraints, Eq. (1) is extended using selection matrices that allow for missing observations and distributed lag matrices that enforce the linear constraints. The ‘extended’ measurement equation is given by

2 $$\begin{aligned} {{\textbf {y}}}_t&= {{\textbf {S}}}_t\ \Big ({{\textbf {L}}}_0 \varvec{\Lambda } {{\textbf {f}}}_t + {{\textbf {L}}}_1 \varvec{\Lambda } {{\textbf {f}}}_{t-1} + \cdots + {{\textbf {L}}}_s \varvec{\Lambda } {{\textbf {f}}}_{t-s} + {{\textbf {e}}}_t\Big ), \qquad {{\textbf {e}}}_t \sim {\mathcal {N}}\left( {{\textbf {0}}}, \varvec{\Sigma }\right) , \end{aligned}$$

where ${{\textbf {S}}}_t$ is a diagonal selection matrix of order $n\times n$, featuring ones on the diagonal if the corresponding value in ${{\textbf {y}}}_t$ is measured and zeros otherwise (see, for instance, Banbura and Modugno 2014). A selection matrix ensures that the factors are only updated when the data is indeed measured. Therefore, this framework easily deals with missing observations due to ragged edges, limited historical availability, and mixed frequencies. An annual output gap, for instance, is only registered in the last quarter of a year and set to zero else. The corresponding entry in ${{\textbf {S}}}_t$ is set to one in the last quarter and zero else, ensuring that both sides of the equation equal zero if the annual output gap is not measured.

The distributed lag matrices ${{\textbf {L}}}_0, \ldots , {{\textbf {L}}}_s$ are also diagonal matrices that impose linear temporal constraints on the factors (Bai and Wang 2015; Stock and Watson 2016). Since annual output gaps are simply an average of the quarterly output gaps, the entries in the distributed lag matrices are either 1, 0, or 0.25. An annual output gap, registered only in the fourth quarter, is given by the average of the factors in the current quarter and the three preceding quarters. A quarterly output gap only loads on the factors in the corresponding quarter.

### State transition

The state equation determines how the hierarchical components interact. It may be, for instance, that small, globalized economies react stronger to changes in the global cycle than large, self-sustaining countries. The global, regional, and local components at time *t* are stacked in a vector ${{\textbf {f}}}_t$. To allow for comovements between and within all levels, it is assumed that ${{\textbf {f}}}_t$ follows a vector autoregressive process according to the state equation

3 $$\begin{aligned} {{\textbf {f}}}_t&= \varvec{\Phi }_1 {{\textbf {f}}}_{t-1} + \cdots + \varvec{\Phi }_p {{\textbf {f}}}_{t-p} + {{\textbf {v}}}_t, \qquad {{\textbf {v}}}_t \sim {\mathcal {N}}\left( {{\textbf {0}}}, \varvec{\Omega }\right) , \end{aligned}$$

where $\varvec{\Phi }_1, \ldots , \varvec{\Phi }_p$ are $q\times q$ matrices of autoregressive coefficients at lags 1 to *p*. We estimate the model using 4 lags, hence $p = 4$. The autoregressive coefficients are restricted to nonnegative values. This assumption improves numerical stability of the sampling procedure and is rarely violated when relaxed since a negative synchronization of business cycles is unlikely. The error term ${{\textbf {v}}}_t$ is normally distributed with mean zero and diagonal covariance matrix $\varvec{\Omega }$. The assumption of uncorrelated innovations in the state equation can be motivated by the fact that common economic shocks at the local level are likely to be captured by a common factor at a higher hierarchical level. In addition, the assumption of a diagonal covariance matrix substantially reduces the computational burden since the inversion of $\varvec{\Omega }$ is straightforward even for large models.^3^

The estimation and interpretation of $\varvec{\Phi }_1, \ldots , \varvec{\Phi }_p$ is demanding due to the large number of autoregressive coefficients involved. To deal with the issue of over-parameterization, we employ a stochastic search variable selection approach that reduces dimensionality by selecting only a few relevant variables (George and McCulloch 1993; Berger et al. 2021). Kuo and Mallick (1998) state that subset selection enables a sparse parameterization, increases the precision of statistical estimates, and allows differentiating between important and negligible predictors. Furthermore, selecting a subset of variables instead of using shrinkage priors (see, for instance, Polson and Scott 2010; Bhattacharya et al. 2016) implies that inconsequential parameters are exactly zero, which facilitates the use of sparse matrix algorithms during model estimation. Berger et al. (2021) use stochastic search to select relevant factors in the measurement equation of a multi-level DFM.

We follow Kuo and Mallick (1998) in embedding indicator variables in the regression equation and rewrite the vector autoregression as in Chan (2019). Defining $vec([\varvec{\Phi }_1, \ldots , \varvec{\Phi }_p]') = \varvec{\Gamma }\varvec{\theta }$, we can express Eq. (3) as

4 $$\begin{aligned} {{\textbf {f}}}_t&= {{\textbf {X}}}_t\varvec{\Gamma } \varvec{\theta } + {{\textbf {v}}}_t, \end{aligned}$$

where ${{\textbf {X}}}_t = {{\textbf {I}}}_q \otimes [{{\textbf {f}}}'_{t-1}, \ldots , {{\textbf {f}}}'_{t-p}]$. $\varvec{\theta }$ contains the correspondingly stacked vector autoregressive coefficients. The diagonal matrix $\varvec{\Gamma }$ contains $pq^2$ binary indicator variables $\gamma _{ijr}$ that follow a Bernoulli distribution with probabilities $\pi _{1,ijr}$. Each entry in ${{\textbf {f}}}_t$ can be expressed equivalent to Eq. (4) as

5 $$\begin{aligned} f_{i,t} = \sum _{j=1}^q \sum _{r=1}^p \gamma _{ijr} \theta _{ijr} f_{j,t-r} + v_{i,t} \end{aligned}$$

by indexing the dependent variable with *i*, the independent variable with *j*, and the lag with *r*. The indicator variable $\gamma _{ijr}$ determines if the predictor is included ($\gamma _{ijr}$ = 1) or excluded ($\gamma _{ijr} = 0$) from the reduced regression model. If $\gamma _{ijr} = 0$, the factor of country *i* is not influenced by the factor of country *j* at lag *r* and the model does not estimate $\theta _{ijr}$. If $\gamma _{ijr} = 1$, $f_{j,t-r}$ influences $f_{i,t}$ and the model estimates the corresponding coefficient $\theta _{ijr}$.

### Spatial Prior Information

In the absence of prior information on how important a predictor is, a prior inclusion probability of 0.5 is typically chosen for all indicators. This reflects a prior belief that all variables are equally likely to be included. However, as O’Hara and Sillanpää (2009) point out, a typical problem with stochastic search variable selection is poor mixing during the sampling process. This issue can be tackled by using informative priors for the probability of a variable being included in the model. As suggested by George and McCulloch (1993), priors can also be used to influence the number of variables included in the model. For output gaps, it is reasonable to form priors based on the assumptions that output gaps are fairly persistent over time and that comovements are usually stronger for geographically close countries and regions. To impose time dependence, we follow the widely used Minnesota prior and assume that own lags as well as more recent lags are more important (Doan et al. 1984; Koop and Korobilis 2009). Regarding the spatial dependence, we follow LeSage and Krivelyova (1999) and LeSage and Cashell (2015) in assuming that neighboring spatial entities exert a stronger influence than non-adjacent entities.^4^ They set the prior mean for adjacent countries to one and for non-adjacent countries to zero. Whereas LeSage and Krivelyova (1999) assume only spatial dependence, LeSage and Cashell (2015) implement spatial as well as time dependence. We extend their procedure by using a stochastic search variable selection approach. This approach allows us to include the spatial information in a straightforward fashion. Instead of a normal prior that requires careful tuning, we only need to specify the prior probability that a coefficient is included in the reduced model. Furthermore, instead of a binary setup based on contiguities, we use a continuous measure based on geographic distances. This gives us a finer mapping of geographic proximity to inclusion probability.

We determine a prior inclusion probability $\pi _{0,ijr}$ for each autoregressive coefficient. Since we have a total of *q* global, regional, and local factors that follow a VAR process of order *p*, there are $pq^2$ binary indicators. We use the following equation to calculate $\pi _{0,ijr}$ for the dependent variable *i* and independent variable *j*

6 $$\begin{aligned} \pi _{0,ijr} = \left( \frac{\exp (-\rho d_{ij})}{\sum _j \exp (-\rho d_{ij})}\right) ^\alpha \frac{1}{r^\beta }, \qquad \text {for } j \ne i \text { and }j, i \ne {\text {world}} \end{aligned}$$

where $d_{ij}$ is the shortest distance between any point on the borders of countries (or regions) *i* and *j*.^5^ Hence, the distance between two neighboring countries is zero. The first fraction in the equation, $\frac{\exp (-\rho d_{ij})}{\sum _j \exp (-\rho d_{ij})}$, transforms the distances $d_{ij}$ into probabilities using the normalized exponential function, also referred to as the softmax function. It accounts for the assumption that the dependence between two geographic entities decreases exponentially with their distance. The normalization ensures that the vector of prior probabilities of an entity *i* sums to one. Note that this vector does not contain the values for the own lag ($j = i$) and the lag of the world factor ($j = {\text {world}}$). Put in other words, the denominator sums over all *j* except for the own and the world factor. We explain in the next two paragraphs how we calculate the prior inclusion probabilities for the own lags and the world factor. The hyperparameter $\rho > 0$ regulates the degree of shrinkage, depending on the distance: The higher $\rho$, the stronger the decay with distance. The minus before $\rho$ ensures that the entities with the shortest geographic distance have a higher prior inclusion probability. The hyperparameter $\alpha > 0$ scales the prior inclusion probabilities, which allows a researcher to influence the number of included variables and, hence, the model size. The second fraction in Eq. (6), $\frac{1}{r^\beta }$, introduces a decay over the lag length *r*. The hyperparameter $\beta > 0$ leads to an exponential decrease of the prior probability with increasing lag length.

The prior inclusion probabilities for the own lags ($j=i$), the world factor as the predictor ($j={\text {world}}$), and all predictors in the equation with the world factor as the dependent variable ($i={\text {world}}$) require special treatment because we cannot build upon distances. For the prior inclusion probabilities of the own lags ($j=i$), we impose the belief that output gaps are relatively persistent over time. We use the following equation

7 $$\begin{aligned} \pi _{0,iir} = \frac{1}{r^\beta } \end{aligned}$$

such that the probability of the variable being included is one for the first lag and decays exponentially with increase in lag length. Similarly, we set the prior probability for the world factor as the predictor variable *j* to $\frac{1}{r^\beta }$. We thereby assume that the lagged world factor is an important predictor for all factors. In the equation with the world factor as the dependent variable *i* and $r=1$, we set the prior for each region *j* to one divided by the number of regions and the prior for each country *j* to one divided by the number of countries. For $r>1$, we divide the values of $r=1$ by $r^\beta$ such that a lag decay is introduced. Consequently, we assume that the temporal dependence decreases with the lag length and that the world factor depends more on the lagged regional factors compared to the lagged country-specific factors.

The $pq^2$-dimensional vector with all prior inclusion probabilities is defined as $\varvec{\pi }_0 = {\text {vec}}([\varvec{\pi }_{0, r=1}, \ldots , \varvec{\pi }_{0, r=p}]')$ where

$$\begin{aligned} \varvec{\pi }_{0, r} = \begin{bmatrix} \pi _{0,i=1,j=1,r} &{}\quad \pi _{0,i=1,j=2,r} &{}\quad \ldots &{}\quad \pi _{0,i=1,j=q,r} \\ \pi _{0,i=2,j=1,r} &{}\quad \pi _{0,i=2,j=2,r} &{}\quad \ldots &{}\quad \pi _{0,i=2,j=q,r} \\ \vdots &{}\quad \vdots &{} &{}\quad \vdots \\ \pi _{0,i=q,j=1,r} &{}\quad \pi _{0,i=q,j=2,r} &{}\quad \ldots &{}\quad \pi _{0,i=q,j=q,r} \\ \end{bmatrix} \quad \text {for }r \in \{1,\ldots , p\}. \end{aligned}$$

With $\alpha = 1$, our specification implies that the sum of each row in $\varvec{\pi }_{0, r=1}$ is three. Consequently, if $p=1$, our prior belief is that in each autoregressive equation three out of the *q* factors have a nonzero regression coefficient. For the regions and countries, this sum of three consists of a probability of one for the lag of the world factor, one for the own lag, and one in total for the remaining countries and regions where the probability depends on the distances. The sum of each row in $\varvec{\pi }_{0, r}$ decreases with increasing *r* as we expect that fewer regression coefficients are different from zero. With $\beta =2$, the sum of each row in $\varvec{\pi }_{0, r=2}$ is equal to 0.75, in $\pi _{0, r=3}$ equal to 0.33, in $\pi _{0, r=4}$ equal to 0.19, and so forth. In ‘Appendix B,’ we provide two examples of the probabilities.

### Estimation

Since ${{\textbf {S}}}_t$ and ${{\textbf {L}}}_0, \ldots , {{\textbf {L}}}_s$ are known ex ante, the computational task reduces to estimating the dynamic factors ${{\textbf {f}}}_t$ and the parameter $\varvec{\Phi }_1, \ldots , \varvec{\Phi }_p$, $\varvec{\Sigma }$, and $\varvec{\Omega }$. We sample the latent states jointly using the efficient and sparse algorithm by Chan and Jeliazkov (2009). To group the parameters in appropriate blocks, we stack the measurement Eq. (2) over the *T* time periods.

8 $$\begin{aligned} {{\textbf {y}}}&= {{\textbf {G}}}{{\textbf {f}}} + {{\textbf {e}}}, \qquad {{\textbf {e}}} \sim {\mathcal {N}}({{\textbf {0}}}, {{\textbf {I}}}_T \otimes \varvec{\Sigma }), \end{aligned}$$

where

$$\begin{aligned} \underset{nT\times 1}{{{\textbf {y}}}}&= \begin{bmatrix} {{\textbf {y}}}_1\\ {{\textbf {y}}}_2\\ \vdots \\ {{\textbf {y}}}_T\\ \end{bmatrix}, \\ \underset{nT\times q(T+s)}{{{\textbf {G}}}}&= \begin{bmatrix} {{\textbf {S}}}_1 {{\textbf {L}}}_s \varvec{\Lambda } &{}\quad \ldots &{}\quad {{\textbf {S}}}_1 {{\textbf {L}}}_0\varvec{\Lambda } &{} &{} &{} \\ &{}\quad {{\textbf {S}}}_2 {{\textbf {L}}}_s \varvec{\Lambda } &{}\quad \ldots &{}\quad {{\textbf {S}}}_2 {{\textbf {L}}}_0 \varvec{\Lambda } &{} &{} \\ &{} &{}\quad \ddots &{} &{}\quad \ddots &{} \\ &{} &{} &{}\quad {{\textbf {S}}}_T {{\textbf {L}}}_s \varvec{\Lambda } &{}\quad \ldots &{}\quad {{\textbf {S}}}_T {{\textbf {L}}}_0 \varvec{\Lambda } \\ \end{bmatrix} . \end{aligned}$$

Correspondingly, the state Eq. (3) is stacked according to

9 $$\begin{aligned} {{\textbf {H}}}{{\textbf {f}}}&= {{\textbf {v}}}, \qquad {{\textbf {v}}} \sim {\mathcal {N}}({{\textbf {0}}}, {{\textbf {I}}}_{T+s} \otimes \varvec{\Omega }), \end{aligned}$$

where

$$\begin{aligned} \underset{q(T+s) \times q(T+s)}{{{\textbf {H}}}}&= \begin{bmatrix} {{\textbf {I}}}_q &{} &{} &{} &{} &{} \\ -\varvec{\Phi }_1 &{}\quad {{\textbf {I}}}_q &{} &{} &{} &{} \\ \vdots &{}\quad \ddots &{}\quad {{\textbf {I}}}_q &{} &{} &{} \\ -\varvec{\Phi }_p &{}\quad \ldots &{}\quad -\varvec{\Phi }_1 &{}\quad {{\textbf {I}}}_q &{} &{} \\ &{}\quad \ddots &{}\quad \ddots &{}\quad \ddots &{}\quad \ddots &{} \\ &{} &{}\quad -\varvec{\Phi }_p &{}\quad \ldots &{}\quad -\varvec{\Phi }_1 &{}\quad {{\textbf {I}}}_q\\ \end{bmatrix}, \qquad \underset{q(T+s) \times 1}{{{\textbf {f}}}} = \begin{bmatrix} {{\textbf {f}}}_{1-s} \\ \vdots \\ {{\textbf {f}}}_{0} \\ {{\textbf {f}}}_{1} \\ \vdots \\ {{\textbf {f}}}_T \\ \end{bmatrix} . \end{aligned}$$

Following Chan and Jeliazkov (2009), the precision matrix ${{\textbf {K}}}$ is given by ${{\textbf {H}}}' ({{\textbf {I}}}_{T+s} \otimes \varvec{\Omega })^{-1} {{\textbf {H}}}$ and the conditional posterior of the factors is normally distributed with

$$\begin{aligned} {{\textbf {f}}} \sim {\mathcal {N}}({{\textbf {p}}}_1, {{\textbf {P}}}_1^{-1}) \quad \text {where} \quad {{\textbf {P}}}_1&= {{\textbf {K}}} + {{\textbf {G}}}'({{\textbf {I}}}_T \otimes \varvec{\Sigma }^{-1}){{\textbf {G}}} \\ {{\textbf {p}}}_1&= {{\textbf {P}}}^{-1} ({{\textbf {G}}}'({{\textbf {I}}}_T \otimes \varvec{\Sigma }^{-1}){{\textbf {y}}} ) . \end{aligned}$$

This algorithm is computationally very efficient if block-banded and sparse matrix algorithms are used.^6^ For further details on the estimation algorithm and the conditional distributions of the remaining parameters, see ‘Appendix A.’

## Global and local cycles

### Data and output gaps

We extract the common factors from a large collection of quarterly and annual output gap estimates for various countries. By combining several models for each country, we account for the fact that the output gap is unobservable and there exists no true estimate (see, for instance, Orphanides and van Norden 2002; Berger et al. 2023). The HP-filter, for instance, has been criticized by Nelson and Plosser (1982), Cogley and Nason (1995), and more recently by Hamilton (2018) for introducing spurious cycles and strongly depending on the smoothing parameters. Pooling several estimates is likely to lead to more robust results, in particular for less developed countries with a more volatile business cycle. In the absence of timely and reliable national accounting data, output gap estimates are usually subject to mixed frequencies, ragged edges, and limited historical availability. Therefore, our approach may also be useful for reliable real-time estimates in developing countries since the global and regional components of their output gaps are based on a broader set of information. We focus on five well-established methods that rely only on real gross domestic product, are applicable to quarterly as well as annual data, and do not require excessive computational effort. Most of these approaches have also been used by Garratt et al. (2014) in their ensemble nowcasts of the output gap.

First, we apply the well-known filter proposed by Hodrick and Prescott (1997), using the parametrization suggested by Ravn and Uhlig (2002). Second, we apply a bandpass filter proposed by Baxter and King (1999). Third, we use the filter proposed by Hamilton (2018), which takes the 2-year forecast error of a projection based on an autoregressive model as the cyclical component of output. Fourth, we apply an unobserved components model following Watson (1986), which performs a decomposition of GDP into a trend with stochastic drift and a cycle that follows a stationary autoregressive process. Lastly, we fit a simple cubic spline to logarithmized GDP. It should be noted that this selection does not necessarily represent the most suitable methods. In particular, the generic parameter assumptions may not be appropriate for every country. To make the results comparable across countries, we choose to omit relational methods, where the output gap is modeled as a function of well observable market outcomes, such as inflation or unemployment (Kuttner 1994; Gerlach and Smets 1999; Graff and Sturm 2012). Due to data limitations, we also do not use output gaps that originate from production function approaches. Table 1 provides an overview of the data by giving the standard deviations of the output gaps by region and estimation method.

**Table 1:** Ex-post variation of output gap estimates

	#	Hodrick–Prescott filter		Baxter–King filter		Hamilton filter		Unobs. components		Cubic spline	
		*a*	*q*	*a*	*q*	*a*	*q*	*a*	*q*	*a*	*q*
Total	157	3.2	2.3	3.1	2.0	8.4	4.3	9.3	3.5	6.2	3.3
Australia and Oceania	3	1.1	1.4	1.0	1.0	2.4	2.3	3.0	2.2	1.7	1.9
North America	2	1.0	1.5	1.0	1.1	2.4	2.5	3.3	2.6	1.8	2.1
Europe	39	2.9	2.5	2.8	2.1	8.2	4.7	9.7	3.5	6.3	3.7
East and Southeast Asia	16	2.2	1.5	2.3	1.4	5.8	2.6	6.0	1.8	4.2	2.1
North Africa and W. Asia	27	4.9	2.7	3.9	2.4	12.0	5.1	15.1	3.9	9.6	3.5
Sub-Saharan Africa	39	3.3	2.7	3.5	1.5	9.1	3.0	8.0	7.9	5.8	2.4
Latin America and Carib	24	2.0	2.4	2.5	1.9	5.7	3.9	4.8	4.0	4.2	2.8
South Asia	7	2.1	3.2	2.3	2.1	4.4	4.5	3.8	4.7	2.8	3.3

Table exhibits standard deviations for annual and quarterly output gap estimates across geographic regions

# indicates the number of countries at each level. Not all countries have quarterly data

We apply the five methods for all available annual and quarterly time series on real gross domestic product. The annual data originates from the Penn World Table (version 10.0) and is available for 154 countries, the quarterly data originates from the Quarterly National Accounts of the OECD and is available for 47 countries. In total, 157 countries are included. The data starts in the first quarter of 1990 and ends in the fourth quarter of 2020. As outlined in Sect. 2.1, our approach can handle a data matrix with missing observations.

**Fig. 1:**
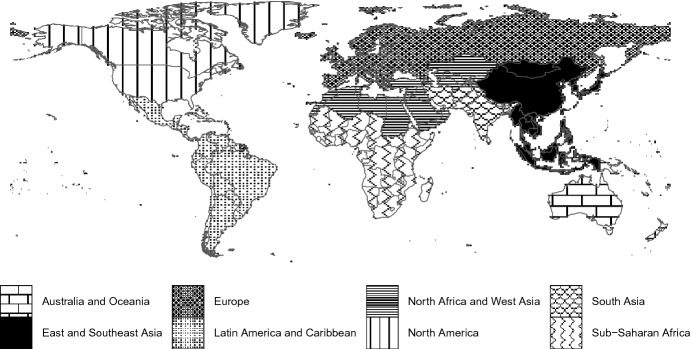
Choropleth map highlighting the eight regional aggregates

We classify all countries into 21 geographic subregions according to the United Nations geoscheme, which is based on the M49 coding classification. We then aggregate these subregions further into eight economic regions. Figure 1 shows a map of the regions and ‘Appendix D’ lists the assignment of the countries to the different regions. These regions are based on regional aggregation schemes used by large international institutions and reflect our belief that they share a common cyclical component.

### Decomposition of output gaps

The multi-level DFM allows us to decompose the (reconciled) output gap of each country into a global, a regional, and a local component. The global factor captures the variation in the output gaps that is common to all countries. The regional factor represents the comovements that are unique to all countries within a specific region. Finally, the local factor captures the country-specific part of the output gap that is not shared with the region and the world as a whole. Since we have restricted the factor loadings to be binary, the estimated factors can be interpreted directly as the contribution of the corresponding hierarchical level to a country’s output gap in percentage points. In order to determine peaks and troughs in these global, regional, and local business cycle fluctuation components, we test whether they are different from zero at a 5% significance level.

**Fig. 2:**
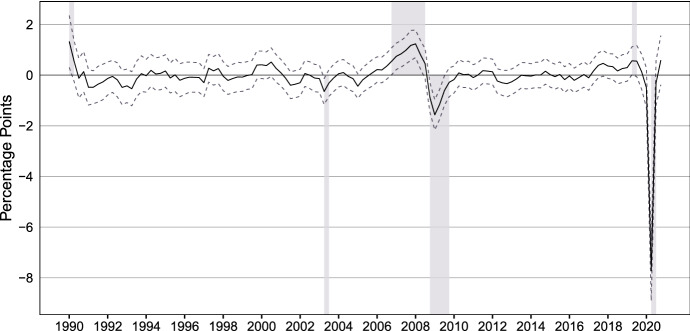
Global component of output gaps. The solid line represents the estimated global factor that measures in percentage points the component of the output gaps that is common to all countries. The dashed lines mark the 95%-confidence interval of the factor. The shaded areas highlight the periods where the factor is significantly different from zero

Figure 2 shows the estimated global factor from 1990 to 2020. The factor is significantly different from zero in several years, indicating the existence of a global component to cyclical fluctuations across countries. Consistent with the findings in Kose et al. (2003), the global cyclical component is fairly persistent with an autoregressive coefficient at the first lag of 0.63. Consistent with findings in Kose et al. (2020), the Asian financial crisis in 1997 and 1998 and the bursting of the tech bubble in 2000 had no significant impact on the global component. The global financial crisis, on the other hand, marked a sharp common decline in capacity utilization in each country. By far the strongest common deviation from potential output was caused by the COVID-19 recession, amounting to a value of more than − 7 percentage points in the second quarter of 2020. Scale and sign of the common cyclical fluctuations correspond to those found in alternative approaches, for instance Berger and Wortmann (2020) and Berger et al. (2021).

**Fig. 3:**
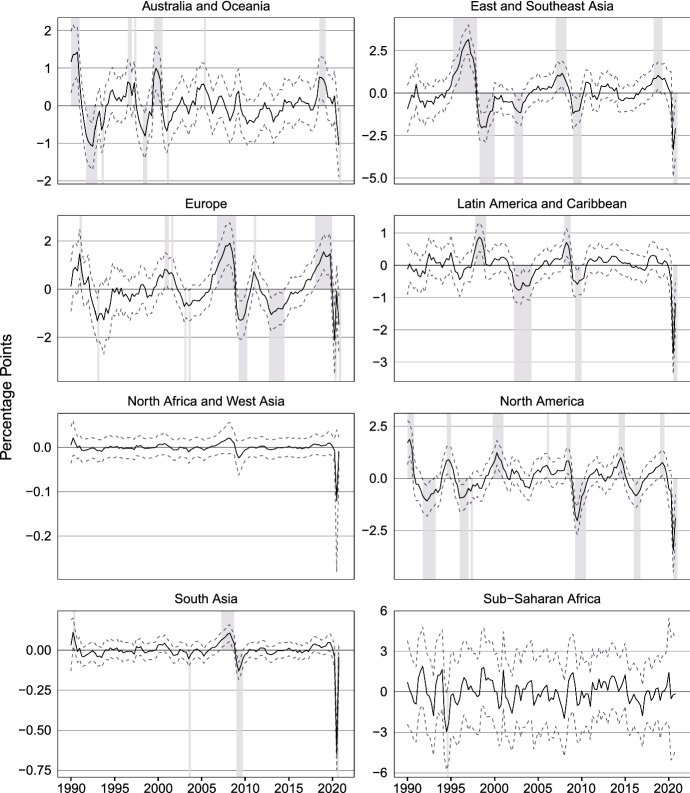
Regional components of output gaps. The solid line represents the estimated regional factors that measures in percentage points the component of the output gaps that are common to all countries within a region. The dashed lines mark the 95%-confidence interval of the factors. The shaded areas highlight the periods where the factor is significantly different from zero

**Fig. 4:**
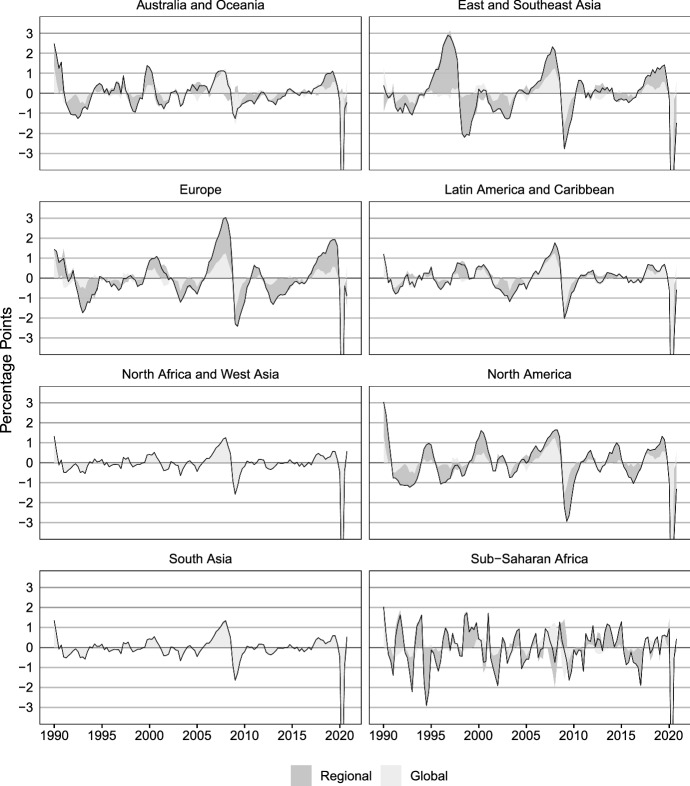
Global and regional components of output gaps. The lighter area represents the global output gap component that is common to all countries. The darker area represents the region-specific component that measure the part of the output gaps that is common to all countries within a region. The black line shows the sum of the two components. Figures are truncated at − 3.5 percentage points to improve readability

Regional components explain the variations in output gaps that are common only to the countries in a specific region. Figure 3 shows the eight regional factors where periods of a significant deviation from zero are highlighted in gray. In Fig. 4, the regional components are shown together with the global component. While there appears to be a significant common component in some regions, others show no sign of a shared cycle. The strongest regional cycles can be found in North America, Europe, and East and Southeast Asia. The cycles in Latin America and Australia are somewhat smaller but still significantly different from zero in many quarters. The factor for Sub-Saharan Africa is highly volatile and has large standard errors such that no significant common component results in almost all quarters. The regional factors for South Asia and North Africa and West Asia are virtually nonexistent. Even though this paper follows a novel approach with restricted factor loadings, the findings are quite similar to results in the related literature. Berger and Wortmann (2020), for instance, also find little comovement in clusters of African or Middle Eastern countries.

It may well be that some declines in output happen simultaneously in multiple regional components. The global financial crisis, for instance, caused very negative output gaps in 2009 in Europe, North America, and East and Southeast Asia, whereas the decline in Sub-Saharan Africa and Australia was only driven by the global factor. The model also identifies several region-specific shocks. For instance, the Asian financial crisis affected in 1998 in particular the output gaps of countries in East and Southeast Asia, the crisis of the European exchange rate mechanism in 1992 and 1993 is mainly reflected in the European cycle, or the South American economic crisis in 2002 and 2003 is visible in the Latin American factor only. Also the COVID-19 recession led to regional differences, although the global component accounts for a large fraction of the declines in capacity utilization. The regional components of Europe, North America, East and Southeast Asia, and Latin America are more negative in 2020 than the other regional components. The components of Sub-Saharan Africa and North Africa and West Asia, on the other hand, are not significantly different from zero in 2020.

**Fig. 5:**
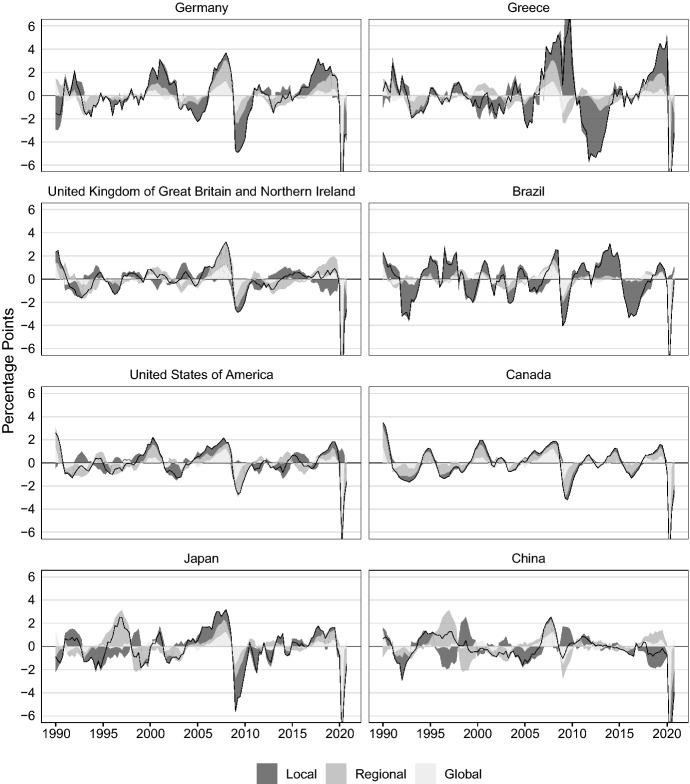
Global, regional, and country-specific components of output gaps The output gaps of the countries decomposed into three parts: A global component (lightest area), a region-specific component (medium dark area), and a country-specific component (darkest area), all in percentage points. The black line shows the estimated output gap in percent given by the sum of the three components. Figures are truncated at − 6 and $+$ 6 percentage points to improve readability

Figur 5  adds the local cycle to show the contributions of all three hierarchical levels. We limit our exposition here to a selection of eight countries that exhibit interesting decompositions. The shaded areas mark the parts of the output gap attributable to the country-specific, regional, and global cycles. The black line represents the sum of the three factors and thereby the reconciled output gap of the country. Figure 5 visualizes that the global factor usually constitutes a substantial portion of the output gap. On average, the global component accounts for 18% of the output gap, the regional factor for 24%, and the country-specific factor for 58% of the absolute sum of the three components. However, these shares vary substantially between the countries. These findings are mostly in line with Kose et al. (2003), where the country-specific factors explain a large fraction of cyclical fluctuations, in particular for small, developing countries.

The estimated components allow us to study the integration of local cycles in the regional and global business cycles. Of particular interest is the development of countries within the same region. In Europe, for instance, Germany recovers relatively quickly following the global financial crisis and is barely affected by the subsequent sovereign debt crisis. The local cycle even contributes to an inflationary output gap in multiple quarters while the regional cycle for Europe indicates undercapacities. Greece, on the other hand, recovers very slowly and the local component remains negative for 3 years. For the UK, a negative factor results in the year 2019, reflecting the economic uncertainties related to the Brexit negotiations. A volatile and strong local cycle can be found for Brazil. 64% of the absolute sum of the three components is attributable to the country-specific factor. Brazil experienced a severe economic crisis in 2014 that was caused by deteriorating commodity prices and, more importantly, a series of unfortunate macroeconomic policies. The persistently negative output gap was, therefore, limited to the Brazilian economy and is not visible in the regional cycle for Latin America. The local cycle for the USA and Canada are relatively small because the regional cycle captures a large part of the cyclical variation. This points toward a strong cyclical integration of the countries included in the corresponding aggregate. Of the three components, the local cycle accounts for only 37% for the USA and for 23% for Canada. The figure for Japan reveals that the Japanese economy was also hit relatively hard by the global financial crisis. Lastly, China was only marginally affected by the global financial crisis but drifted into a persistent undercapacity in recent years. The Chinese factor is negatively correlated with the sum of the two components of the corresponding higher hierarchical levels—the East and Southeast Asian and the global component. Put in other words, the cyclical phases of China do not coincide at times with the phases of the remaining East and Southeast Asian countries. Given China’s large domestic market and the political heterogeneity of the East and Southeast Asia region, this low degree of synchronization is expected.

In addition, it is instructive to look at the correlations between global, regional, and local cycles. Table 2 gives the correlations between the global and regional factors. We find, for instance, a relatively high correlation between the Latin American and the North American factor, suggesting a comovement of their business cycles. The European cycle exhibits the highest correlation with the global cycle. This indicates that these countries are highly integrated in the global economy.

**Table 2:** Correlation matrix global and regional factors

	(1)	(2)	(3)	(4)	(5)	(6)	(7)	(8)	(9)
(1) World	1.00								
(2) Australia and Oceania	0.04	1.00							
(3) East and Southeast Asia	0.13	0.30	1.00						
(4) Europe	0.47	0.20	0.24	1.00					
(5) Latin America and Carib	0.24	$-$ 0.07	0.29	0.33	1.00				
(6) North Africa and W. Asia	0.26	0.19	0.41	0.40	0.73	1.00			
(7) North America	0.30	0.44	0.17	0.45	0.54	0.68	1.00		
(8) South Asia	0.26	0.18	0.41	0.38	0.72	1.00	0.67	1.00	
(9) Sub-Saharan Africa	$-$ 0.05	$-$ 0.10	$-$ 0.12	$-$ 0.15	0.01	$-$ 0.06	$-$ 0.06	$-$ 0.06	1.00

Table shows correlations between global and regional output gap components

The estimated autoregressive coefficients of the factors are informative as well since they explain which cycles explain or predict other cycles. Our results suggest, for instance, that the South Korean cycle lags the Japanese cycle, the Uruguayan lags the Argentinian cycle, and the Taiwanese cycle lags the Chinese cycle.

The coefficients for the world factor as the explanatory variable differ highly between the countries and regions. For instance, the factors of Germany, China, and North America depend highly on the first lag of the world factor, where the value of the coefficients is between 0.3 and 0.6. Other cycles, such as those of the region Australia and Oceania, do not depend on the lagged world factor. The own lag is in most equations the most important determinant, reflecting the findings in Kose et al. (2003). On average, the coefficient for the own lag is 0.3 which indicates some persistence of the cycles. We obtain, among others, a high own lag coefficient for Germany (0.8) and the USA (0.5).

## Conclusion

We have proposed a multi-level dynamic factor model to identify common and country-specific components in output gap estimates. Our model combination framework pools information from several output gap estimation methods, which may be subject to mixed frequencies, random patterns of missing observations, and ragged edges. Appropriate restrictions on the factor loadings impose a hierarchical multi-level factor structure such that each output gap estimate consists of a global, a regional, and a local component, as well as an idiosyncratic component that is specific to each model and country.

We contribute to the literature on the identification of global and regional business cycles by providing a decomposition of a country’s output gap into common hierarchical components. This information is useful to fiscal and monetary policy makers that implement countercyclical policies at different levels of government. Confirming the findings of Berger et al. (2021), the results provide significant evidence for the existence of a common global cycle and various regional cycles, in particular in North America, Europe as well as East and Southeast Asia. The results suggest that, on average, 18% of the output gap is attributable to the global cycle, 24% to the regional cycle, and 58% to the local cycle. In particular for countries in South Asia and North Africa and West Asia, the local cycle is dominant.

Assuming that fiscal and monetary policy makers aim to reduce cyclical fluctuations as part of their mandate, our model allows us to determine the hierarchical level of international cooperation at which countercyclical policy action would have been appropriate. We argue, for instance, that substantial cooperation of monetary and fiscal authorities had been beneficial during the global financial crisis and the coronavirus pandemic since the global component has been the dominant driver of output gap estimates during both of these events. Countries in Europe, North America as well as East and Southeast Asia exhibit strong common cyclical patterns, which indicates that policy action at regional instead of local levels would be beneficial. Countries in Africa and West and South Asia, on the other hand, show little to no common regional variation, which requires also less international cooperation in mitigating economic shocks.

At an econometric level, we show that spatial information can be used to reduce model complexity not only using shrinkage priors, in line with LeSage and Krivelyova (1999), but also using stochastic search variable selection. In our case, the prior inclusion probabilities are estimated based on the geographic distances between spatial entities. We provide evidence on suitable tuning parameters and show how to determine important predictors in large vector autoregressions. Our approach is more parsimonious in terms of hyperparameters and the resulting posterior coefficients are straightforward to interpret as inclusion probabilities.

## Figures and Tables

**Fig. 6:**
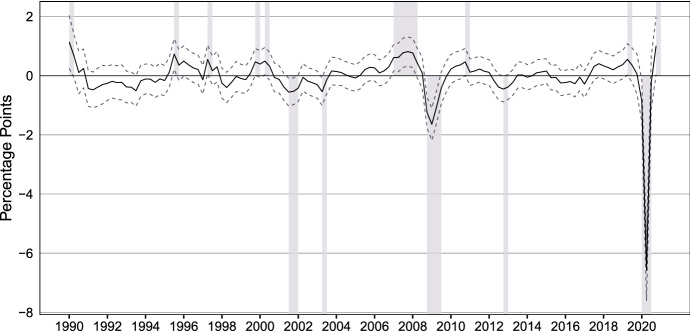
Global component of output gaps with trade prior. The solid line represents the estimated global factor that measures in percentage points the component of the output gaps that is common to all countries. The dashed lines mark the 95%-confidence interval of the factor. The shaded areas highlight the periods where the factor is significantly different from zero

**Fig. 7:**
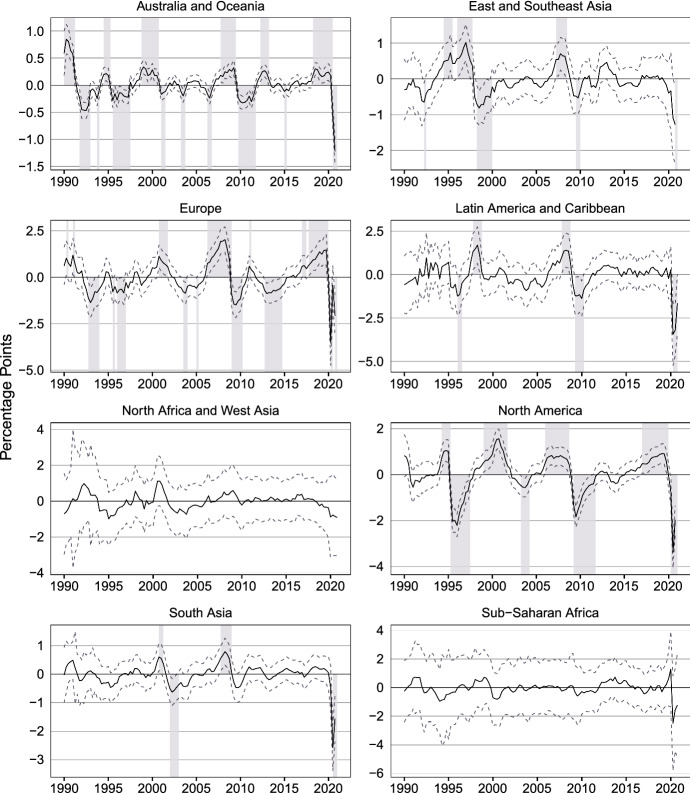
Regional components of output gaps with trade prior. The solid line represents the estimated regional factors that measures in percentage points the component of the output gaps that are common to all countries within a region. The dashed lines mark the 95%-confidence interval of the factors. The shaded areas highlight the periods where the factor is significantly different from zero

**Fig. 8:**
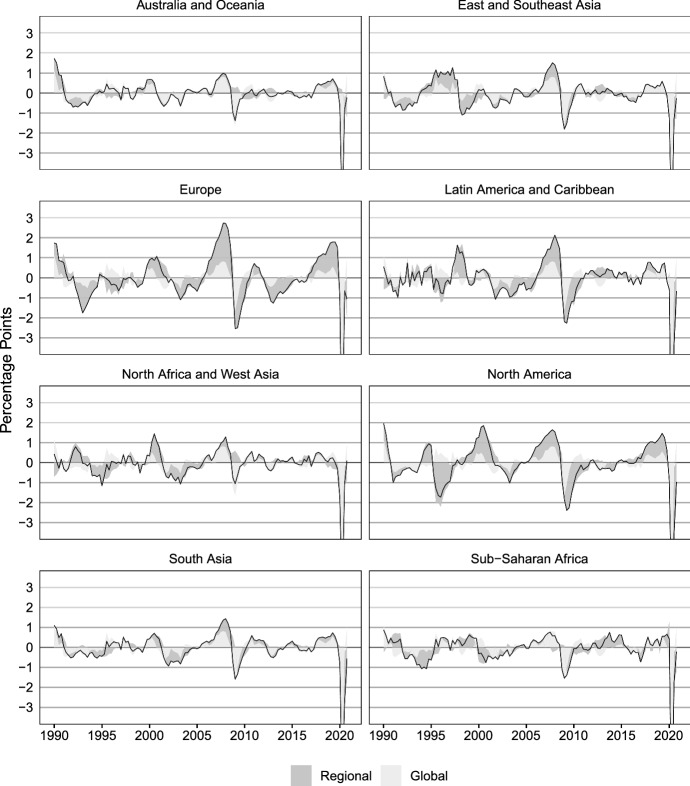
Global and regional components of output gaps with trade prior. The lighter area represents the global output gap component that is common to all countries. The darker area represents the region-specific component that measure the part of the output gaps that is common to all countries within a region. The black line shows the sum of the two components. Figures are truncated at − 3.5 percentage points to improve readability

**Fig. 9:**
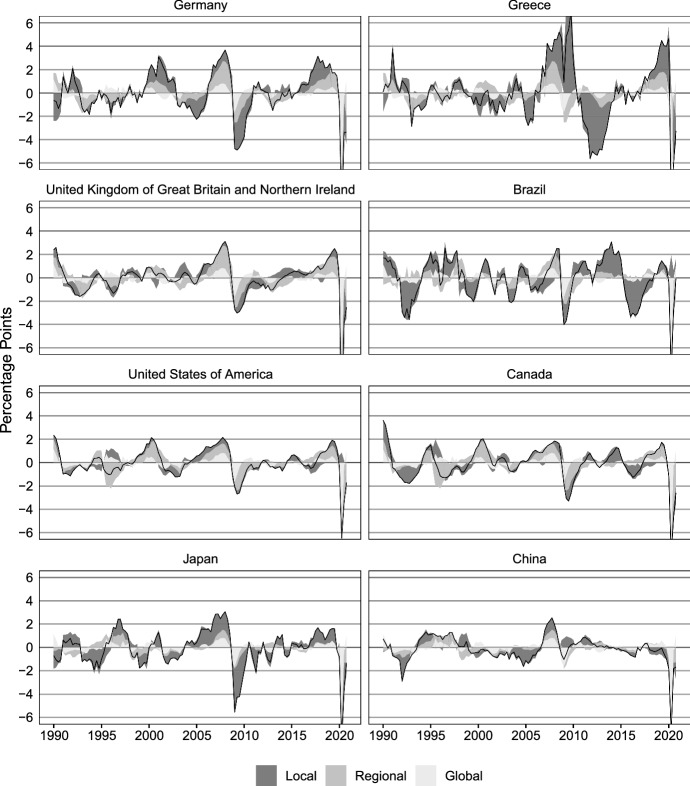
Global, regional, and country-specific components of output gaps with trade prior. The output gaps of the countries decomposed into three parts: A global component (lightest area), a region-specific component (medium dark area), and a country-specific component (darkest area), all in percentage points. The black line shows the estimated output gap in percent given by the sum of the three components. Figures are truncated at − 6 and $+$ 6 percentage points to improve readability

## Appendix A Estimation algorithm

We estimate the model parameters $\varvec{\Gamma }$ and $\varvec{\theta }$ using the Metropolis–Hastings algorithm and the model parameters $\varvec{\Sigma }$ and $\varvec{\Omega }$ as well as the factors ${{\textbf {f}}}_t$ using Gibbs sampling. We discard the first 10,000 draws as burn-in and then save every fifth draw until we have a sample of 5000 draws from the joint posterior distribution. The factor loadings $\varvec{\Lambda }$ are assumed to be fixed.^7^

To estimate the vector autoregressive coefficients $\varvec{\Phi }_1, \ldots , \varvec{\Phi }_p$, it is useful to induce sparsity into the regression. We use indicator variable selection by defining $\varvec{\Gamma }\varvec{\theta } = vec([\varvec{\Phi }_1, \ldots , \varvec{\Phi }_p]')$, where the $pq^2$-dimensional vector $\varvec{\theta }$ holds the unknown regression coefficients. The diagonal matrix $\varvec{\Gamma }$ contains $pq^2$ binary indicators $\gamma _{ijr}$ that determine whether the coefficients are included in the model. Recall the state Eq. (4) from Sect. 3.

10 $$\begin{aligned} {{\textbf {f}}}_t&= {{\textbf {X}}}_t\varvec{\Gamma } \varvec{\theta } + {{\textbf {v}}}_t, \qquad {{\textbf {v}}}_t \sim {\mathcal {N}}\left( {{\textbf {0}}}, \varvec{\Omega }\right) \end{aligned}$$

with ${{\textbf {X}}}_t = {{\textbf {I}}}_q \otimes [{{\textbf {f}}}'_{t-1}, \ldots , {{\textbf {f}}}'_{t-p}]$. If an indicator is equal to zero, the corresponding column in ${{\textbf {X}}}\varvec{\Gamma }$ is set to zero (see, for instance, Kuo and Mallick 1998; Korobilis 2013, for similar approaches). Following Chan (2019), we stack Eq. (10) over each time period into ${{\textbf {x}}} = [{{\textbf {f}}}'_{1+p-s}, \ldots , {{\textbf {f}}}'_T]'$ and ${{\textbf {X}}} = [{{\textbf {X}}}'_{1+p-s}, \ldots , {{\textbf {X}}}'_{T}]'$.

11 $$\begin{aligned} {{\textbf {x}}}&= {{\textbf {X}}} \varvec{\Gamma }\varvec{\theta } + {{\textbf {v}}}, \quad {{\textbf {v}}} \sim {\mathcal {N}}\left( {{\textbf {0}}}, {{\textbf {I}}}_{T-p+s} \otimes \varvec{\Omega } \right) . \end{aligned}$$

While it is possible to sample directly from the conditional posterior distributions of $\varvec{\Gamma }$ and $\varvec{\theta }$, it is computationally more efficient to use a Metropolis algorithm. We simulate candidate values for $\varvec{\Gamma }$ and $\varvec{\theta }$, using appropriate proposal distributions, and evaluate them for each dependent variable in the VAR. The acceptance probability after burn-in is typically around 30%.

The indicator variables $\varvec{\Gamma }$ are sampled using an independence chain Metropolis–Hastings algorithm. We use the prior inclusion probabilities, given by the $pq^2$-dimensional vector $\varvec{\pi }_0$, as proposal distribution. We sample each proposed indicator from a Bernoulli distribution according to

$$\begin{aligned} \gamma _{ijr, \textrm{new}} \sim {\mathcal {B}}(\pi _{0,ijr}) \end{aligned}$$

and construct a proposal indicator matrix $\varvec{\Gamma }_{\textrm{new}}$. Using the indicator matrix $\varvec{\Gamma }_{\textrm{old}}$ from the previous iteration and the proposal indicator matrix $\varvec{\Gamma }_{\textrm{new}}$, we then evaluate the likelihood ratio for each variable $i = 1, \ldots , q$. This is feasible since we assume the errors in the state equation to be uncorrelated. For each $i = 1, \ldots , q$, we determine the acceptance ratio

$$\begin{aligned} R_i&= \frac{\exp \big (-\frac{1}{2\omega ^2_i} ({{\textbf {x}}}_i - {{\textbf {X}}}_i \varvec{\Gamma }_{i, \textrm{new}}\varvec{\theta }_i\big )'\big ({{\textbf {x}}}_i - {{\textbf {X}}}_i \varvec{\Gamma }_{i, \textrm{new}}\varvec{\theta }_i)\big )}{\exp \big (-\frac{1}{2\omega ^2_i} \big ({{\textbf {x}}}_i - {{\textbf {X}}}_i \varvec{\Gamma }_{i, \textrm{old}}\varvec{\theta }_i\big )'\big ({{\textbf {x}}}_i - {{\textbf {X}}}_i \varvec{\Gamma }_{i, \textrm{old}}\varvec{\theta }_i\big )\big )}, \end{aligned}$$

where ${{\textbf {x}}}_i$ and ${{\textbf {X}}}_i$ are the rows corresponding to variable *i*. We then accept the indicators for variable *i* with probability $\min (1,R_i)$ and construct the indicator matrix $\varvec{\Gamma }$.

For the vector autoregressive coefficients $\varvec{\theta }$, we use a random-walk chain Metropolis–Hastings algorithm. Each element in $\varvec{\theta }$ is proposed using

$$\begin{aligned} \theta _{ijr, \textrm{new}} = \theta _{ijr, \textrm{old}} + \xi , \end{aligned}$$

where $\xi \sim {\mathcal {N}}(0,0.05)$. Similar to the indicator variables, we evaluate the acceptance ratio for each dependent variable. It might be useful in some cases to enforce stationarity or positivity of the coefficients. In these cases, one simply discards proposed values that do not comply with these criteria during sampling. Furthermore, it increases the numerical stability of the sampler to include a Minnesota-type prior distribution to shrink coefficients at distant lags toward zero.

We assume all nonzero elements in the factor loadings matrix $\varvec{\Lambda }$ to be equal to one. It greatly speeds up computation to preallocate a sparse matrix and not update it during the sampling procedure. Fixing $\varvec{\Lambda }$ also solves the rotational problem associated with dynamic factor models. In order to identify the dynamic factors, it is necessary to impose restrictions on the factor loadings. It is feasible to put informative priors on the nonzero elements in $\varvec{\Lambda }$ in order to solve the scale, sign, and rotational indeterminacies (see, for instance, Drèze and Richard 1974).

Both $\varvec{\Omega }$ and $\varvec{\Sigma }$ are restricted to be diagonal matrices. It is, therefore, assumed that the dynamic factors account for the cross-correlation in the data and the measurement errors across variables and estimation methods are independent.

$$\begin{aligned} \varvec{\Omega }&= \begin{bmatrix} \omega _1^2 &{} &{} &{} &{}\\ &{}\quad \ddots &{} &{} &{} \\ &{} &{}\quad \omega _i^2 &{} &{}\\ &{} &{} &{}\quad \ddots &{} \\ &{} &{} &{} &{}\quad \omega _q^2 \\ \end{bmatrix}, \quad \varvec{\Sigma } = \begin{bmatrix} \sigma _1^2 &{} &{} &{} &{}\\ &{}\quad \ddots &{} &{} &{} \\ &{} &{}\quad \sigma _k^2 &{} &{}\\ &{} &{} &{}\quad \ddots &{} \\ &{} &{} &{} &{}\quad \sigma _n^2 \\ \end{bmatrix}. \end{aligned}$$

We draw the diagonal elements $\sigma _k^2$ equation-by-equation from an inverse Gamma distribution. The measurement errors ${{\textbf {e}}}_k$ for the *k*th variable can be retrieved from Eq. (2) using ${{\textbf {y}}} - {{\textbf {G}}}{{\textbf {f}}}$. Since missing observations and low-frequency variables lead to zeros in the error term, the shape parameter of the inverse gamma distribution is determined by the number of nonzero elements in ${{\textbf {e}}}_k$, given by $\tau$. The conditional posterior distribution is then given by

$$\begin{aligned} \sigma _k ^2\sim {{\mathcal {I}}}{{\mathcal {G}}}\left( c_{1,k}/2, d_{1,k}/2\right) \quad \text {where} \quad c_{1,k}&= c_{0,k} + \tau \\ d_{1,k}&= d_{0,k} + {{\textbf {e}}}_k {{\textbf {e}}}_k', \end{aligned}$$

where $\tau$ is the number of nonzero elements in the corresponding time series. The priors are chosen to be uninformative by setting $c_{0,k} = 3$ and $d_{0,k} = 1$.

The covariance matrix of the errors in the state equation, given by $\varvec{\Omega }$, is assumed to be diagonal because the innovations to each factor are independent and comovements are accounted for by the common factors. As a result, we draw the diagonal elements $\omega _i^2$ also equation-by-equation from an inverse Gamma distribution. The measurement errors ${{\textbf {v}}}_i$ for the *i*th factor can be retrieved from Eq. (11) using ${{\textbf {x}}} - {{\textbf {X}}} \varvec{\Gamma }\varvec{\theta }$. The conditional posterior distribution is then given by

$$\begin{aligned} \omega _i \sim {{\mathcal {I}}}{{\mathcal {G}}}\left( l_{1,i}/2, m_{1,i}/2\right) \quad \text {where} \quad l_{1,i}&= l_{0,i} + T + s - p\\ m_{1,i}&= m_{0,i} + {{\textbf {v}}}_i {{\textbf {v}}}_i'. \end{aligned}$$

The priors are chosen to be uninformative by setting $l_{0,i} = 3$ and $m_{0,i} = 1$, leaving the smoothness of the common components up to the underlying data.

Unlike in factor models estimated using principal components, orthogonal factors do not arise naturally in DFMs which have been estimated using the Kalman filter. While performing an ex-post orthogonalization of the estimated factors is certainly feasible, the interpretation of the dynamic factors as output gap components is lost in the process. The benefits of doing so are, therefore, likely to be limited to cases where researchers use the orthogonalized factors as input into further models, such as Philips curve estimations.

## Appendix B Specification of spatial prior

We use the following parameters to calculate the spatial priors in Eq. (6): $\rho = 0.01, \beta = 2$, and $\alpha = 1$. To check for the robustness of the results, we evaluated the model with different specifications of these parameters. The changes to the results are qualitatively negligible.

To illustrate the prior inclusion probabilities, we provide two examples. For the German factor as the dependent variable, the prior inclusion probabilities $\pi _{0,i=DE,j,r=1}$ for the first lagged factors are the following: Germany and global cycle (each 1). European regional cycle, France, Luxembourg, Denmark, Switzerland, Poland, Netherlands, Austria, Belgium, Czech Republic (each about 0.09), Italy (0.05), Sweden (0.04), Slovenia (0.02), and Croatia (0.01). These probabilities sum to three. The prior inclusion probabilities for the remaining factors are close to zero. For the USA as the dependent variable, the nonzero prior inclusion probabilities $\pi _{0,i=US,j,r=1}$ for the first lagged factors are the following: USA and global cycle (each 1). North American and Latin American regional cycles, Mexico, Canada (each 0.2), Russia and European regional cycle (each 0.07), and Bahamas (0.06). To obtain the probabilities for $r>1$, the probabilities of $r=1$ are divided by $r^\beta$. As a consequence, the prior inclusion probabilities for the second lag of the world factor as well as for the second own lag are 0.25 ($1/r^\beta$). The probabilities of the remaining second lags sum up to 0.25. Therefore, with $p=2$ the prior specification implies that we expect in each autoregressive equation 3.75 factors with a nonzero regression coefficient. With $p=3$, the corresponding sum is 4.08 and with $p=4$ 4.27.

For the world factor as the dependent variable, the prior inclusion probabilities $\pi _{0,i=WD,j,r=1}$ for the first lagged factors are one for the own lag, one in sum for all regions, and one in sum for all countries. With eight regions and 157 countries, the (rounded) prior inclusion probabilities are 0.13 for each regional cycles and 0.01 for each country. The probabilities are shrunk toward zero with increase in lag length.

## Appendix C Specification of trade prior

To check for robustness of the prior specification $\gamma _{ijr, \textrm{new}} \sim {\mathcal {B}}(\pi _{0,ijr})$, we also tested our model with a prior specification based on trade data instead of spatial data. We used data from the IMF Direction of Trade Statistics (DOTS) that contains bilateral merchandise trade in US Dollars between countries.^8^ We calculate for each country pair the yearly sum of exports and imports and take the mean of the years 2010 to 2019. In addition, we aggregate the bilateral trade volumes to the global and the regional levels for all regions and countries to get, for instance, the trade volume between a country and a region. We calculate for all country and region pairs trade shares and use these shares as the prior inclusion probabilities. As an example, the prior inclusion probabilities $\pi _{0,i=US,j,r=1}$ for the factor of the USA as the dependent variable and the first lagged factors are the following: USA, global cycle (both 1), East and Southeast Asian (0.3), Latin America (0.22), Europe (0.21), Canada (0.17), North America (0.17), Mexico (0.15), Canada (0.14), and so forth.^9^ These probabilities sum to four. In contrast to the spatial priors, the total sum of the prior per country is therefore larger because the regions are not aggregated together with the countries, and therefore, the prior inclusion probabilities for all regions sum to unity with the trade priors. For the regional factors and the world factor as the dependent variable, the prior inclusion probabilities for the first lagged factors are also calculated based on trade shares. For instance, Europe accounts for 39% of the global trade volume among the regions; hence, the prior inclusion probability for Europe when the global factor is the dependent variable is 0.39. To obtain the probabilities for $r>1$, the probabilities of $r=1$ are divided by $r^\beta$.

In Figs. 6, 7, 8, and 9, we show the estimated factors and decompositions when trade priors are used. The results are in general not very different from the results presented in the main section of this paper. However, there are some minor differences.

The results using a trade-based prior suggest a somewhat stronger global cycle leading to, for instance, a significantly negative gap in 2001 (see Fig. 6). Then again, the global cycle estimated with spatial priors is stronger at certain times, for example, at the beginning of the COVID-19 recession in 2020 Q2. The regional cycles for Europe, Latin America, and North America are very similar (see Figs. 7 and 8). The East and Southeast Asian cycle is more pronounced with spatial priors. The Australian cycle is larger with a spatial prior but seems to be more precisely estimated with trade priors. The South Asian cycle is much smaller with spatial priors. However, the significant periods coincide in most instances. The North African and West Asian cycle is larger with trade prior, but in both cases, the cycle is insignificant in all quarters. The Sub-Saharan African cycle is slightly larger and more precisely estimated with trade priors, but the precision is very low with both types of priors.

Since the regional and global cycles differ in parts between the two priors, there are also some differences in the local cycles. With trade priors, the Canadian cycles is larger and the Chinese cycle is smaller (see Fig. 9). Apart from these two countries, the other country-specific cycles shown in Fig. 9 are very similar. On average, the shares of the three levels are not very different between the two priors. On average, the global component accounts for 20% of the output gap (spatial prior: 18%), the regional factor for 24% (spatial prior: 24%), and the country-specific factor for 56% (spatial prior: 58%) of the absolute sum of the three components.

## Appendix D List of countries

*Australia and Oceania (3)*

Australia, Fiji, New Zealand

*East and Southeast Asia (16)*

Brunei Darussalam, Cambodia, China, China, Hong Kong Special Administrative Region, China, Macao Special Administrative Region, Indonesia, Japan, Malaysia, Mongolia, Myanmar, Philippines, Republic of Korea, Singapore, Taiwan (Province of China), Thailand, Viet Nam

*Europe (39)*

Albania, Austria, Belarus, Belgium, Bosnia and Herzegovina, Bulgaria, Croatia, Czechia, Denmark, Estonia, Finland, France, Germany, Greece, Hungary, Iceland, Ireland, Italy, Latvia, Lithuania, Luxembourg, Malta, Moldova (Republic of), Montenegro, Netherlands, North Macedonia, Norway, Poland, Portugal, Romania, Russian Federation, Serbia, Slovakia, Slovenia, Spain, Sweden, Switzerland, Ukraine, United Kingdom of Great Britain and Northern Ireland

*Latin America and Caribbean (24)*

Argentina, Bahamas, Bolivia (Plurinational State of), Brazil, Cayman Islands, Chile, Colombia, Costa Rica, Dominican Republic, Ecuador, El Salvador, Guatemala, Haiti, Honduras, Jamaica, Mexico, Nicaragua, Panama, Paraguay, Peru, Suriname, Trinidad and Tobago, Uruguay, Venezuela (Bolivarian Republic of)

*North Africa and West Asia (27)*

Algeria, Armenia, Azerbaijan, Bahrain, Cyprus, Egypt, Georgia, Iraq, Israel, Jordan, Kazakhstan, Kuwait, Kyrgyzstan, Morocco, Oman, Palestine, State of, Qatar, Saudi Arabia, Sudan, Syrian Arab Republic, Tajikistan, Tunisia, Turkey, Turkmenistan, United Arab Emirates, Uzbekistan, Yemen

*North America (2)*

Canada, USA

*South Asia (7)*

Bangladesh, Bhutan, India, Iran (Islamic Republic of), Maldives, Nepal, Pakistan

*Sub-Saharan Africa (39)*

Angola, Benin, Botswana, Burkina Faso, Burundi, Cabo Verde, Cameroon, Central African Republic, Chad, Congo, Congo (Democratic Republic of the), Côte d’Ivoire, Djibouti, Equatorial Guinea, Eswatini, Ethiopia, Gabon, Gambia, Ghana, Guinea, Kenya, Madagascar, Malawi, Mali, Mauritania, Mauritius, Mozambique, Namibia, Niger, Nigeria, Rwanda, Senegal, Sierra Leone, South Africa, Tanzania (United Republic of), Togo, Uganda, Zambia, Zimbabwe

## Footnotes

1 If a set of estimates is not available or desirable, it might be more appropriate to apply a joint modeling framework where the output gap is estimated directly in the measurement equation. Such a framework has been suggested, for instance, by Huber et al. (2020) or Berger et al. (2023).

2 The factors can be interpreted as a ‘reconciled’ measure of the output gap. Similar state space approaches have been used to reconcile GDP estimates, for instance, by Aruoba et al. (2016) and Jacobs et al. (2020).

3 To relax the assumption of uncorrelated innovations and perform the estimation with a full covariance matrix $\varvec{\Omega }$, it might be feasible to transform the VAR to have a diagonal error covariance matrix as in Carriero et al. (2019) or Koop et al. (2019), which allows for independence across equations and hence a faster equation-by-equation estimation of the coefficients in the state equation.

4 Geographic distances have the advantage that they are readily available for all economic and political units as well as aggregates. However, other bilateral measures such as traffic, political ties, or trade volume between two countries certainly also determine whether two countries exhibit a common cyclical movement. Such measures could provide alternative ways to define the priors. As a robustness check, we provide and discuss results based on bilateral merchandise trade in ‘Appendix C.’

5 We transform the coordinates of the polygons with the Plate Carree projection, an equidistant cylindrical method.

6 It is faster to compute the banded Cholesky factor of ${{\textbf {P}}}_1$ and solve for ${{\textbf {p}}}_1$ by forward- and backward substitution.
